# Socioeconomic status in neurological disorders: a modifiable risk factor?

**DOI:** 10.1007/s00415-022-11123-w

**Published:** 2022-04-25

**Authors:** J. Hrastelj, N. P. Robertson

**Affiliations:** 1 Department of Neurology, University Hospital of Wales, Heath Park, Cardiff, CF14 4XN UK; 2 Department of Neurology, Division of Psychological Medicine and Clinical Neuroscience, Cardiff University, University Hospital of Wales, Heath Park, Cardiff, CF14 4XN UK

## Introduction

Socioeconomic status (SES) is known to play a substantial role in health outcomes and disease frequency, although the bulk of current evidence is derived from vascular disease, infectious diseases, and cancer. More recently, the impact of SES on non-vascular neurological disease has been an increasing focus for researchers with implications for health care providers, government policy, clinical study design, as well as individual patient care. Although SES has been historically considered a risk factor with limited scope for modification at an individual level, recent innovative social policy pilot studies have contested this. Such interventions are highly political, but may bring about novel ways of improving patient outcomes.

The first paper discussed this month is a large epidemiological study of incident cases of epilepsy in children under three years of age in Scotland. The second paper identifies differences in MRI measures of neurodegeneration in cohorts of African Americans and Caucasian individuals. The final paper discusses initial results from a randomised trial of an unconditional cash transfer to new mothers.

## Early childhood epilepsies: epidemiology, classification, aetiology, and socioeconomic determinants

It has been previously observed that epilepsy is more common in lower SES groups. Epilepsy in early childhood is often refractory to treatment, frequently associated with significant developmental problems and has a lasting impact on life trajectory. Symonds et al. conducted a 3-year prospective cohort study of the entire population of Scotland in 2014–2017. They recruited a total of 390 children from all paediatric neurology centres in Scotland. Socioeconomic and clinical data were obtained and all children with refractory epilepsy of uncertain cause were offered whole genome sequencing. Participants were classified into socioeconomic quintiles of the Scottish Index of Multiple Deprivation based on their resident postcode at the time of the first seizure.

The study identified a significant association between SES and epilepsy incidence. Epilepsy incidence in the most deprived quintile was 301 per 100,000 births, compared with 182 per 100,000 in the least deprived quintile. Aetiology was identified in 54% of children and a genetic cause was found in 31%. The gradient in epilepsy incidence with SES was not observed in the group with an identified aetiology, suggesting an increase in multifactorial risk in lower SES groups.

*Comment:* Whilst providing a detailed snapshot of the incidence of epilepsy in young children, this study also demonstrated a clear association with SES. As expected, lower SES was associated with a higher risk of childhood epilepsy, but this was only true for cases with multifactorial aetiology. Therefore, the authors conclude that the increased incidence of epilepsy was not explained by a higher frequency of genetic or structural abnormalities in lower SES groups. The authors also conclude that SES is likely to have had a causal effect on epilepsy incidence because the studied cohort was very young, so that SES was unlikely to have been affected by epilepsy and its associated factors. Exploration of multifactorial risk factors, such as polygenic risk, early life stress, maternal smoking etc., and their relationship with SES will have implications for individual medical and social care, as well as government policy for early years development and support.

Symonds et al. (2021) Brain 144(9):2879–91.

## Socioeconomic status mediates racial differences seen using the AT (N) framework

Alzheimer’s disease (AD) is more common in African Americans than Caucasian populations. This difference has been suggested to be related to a combination of genetic and environmental factors. There have been recent calls for greater exploration of social determinants of health in dementia research. This study took MRI measures of neurodegeneration in cognitively normal African Americans (*n* = 131) and those of Caucasians (*n* = 685) within a longitudinal study of memory and aging. MRI measures included volume of cortical areas most affected by AD, PET imaging of amyloid and tau deposition and functional MRI representing connectivity. SES was measured using the Area Deprivation Index (ADI), which estimates SES for individual zip codes based on 17 metrics. Measures of small vessel disease, cardiovascular disease, blood pressure and BMI were also included in the analyses. Age, sex distribution and frequency of *APOE ε4* allele did not differ between the groups.

In-keeping with previous literature, African Americans had decreased cortical volumes, suggesting greater neurodegeneration despite being asymptomatic. African Americans had significantly lower SES measured by the ADI, higher blood pressure, higher BMI and more white matter T2 hyperintensities. ADI was the only factor identified to have an association with MRI measures and accounted for 11.6% of the difference in cortical volumes.

*Comment*: This study supports previous literature demonstrating that differences in cortical volumes between races can be at least partially explained by SES. There were no differences in amyloid and tau burden or functional MRI measures of connectivity. The most common measure of SES used in dementia studies is the number of years in education, but this explained none of the difference in cortical volumes in this study. The authors conclude that number of years in education is, consequently, an inappropriate measure of SES. The authors also suggest that further exploration of modifiable aspects of SES could yield interventions that could improve outcomes.

Meeker et al. (2020) Ann Neurol 89(2):254–65.

## The impact of a poverty reduction intervention on infant brain activity

Poverty in childhood has been associated with worse neurodevelopmental, educational and health outcomes. It has been difficult to ascertain whether these are mere associations or if there is a causal relationship. As an infant develops, there are characteristic changes measured by electroencephalography (EEG), including a progression from a predominance of low frequency oscillations to mid- and high-frequency oscillations. Earlier progression in these EEG measures has been associated with better cognitive and behavioural outcomes. It is thought that neuroplastic changes in response to environmental stimuli are responsible for these changes. The Baby’s First Years study is the first randomised controlled trial of poverty reduction on early childhood development. One thousand matched low-income mother-infant pairs were randomised to receive either a large ($333/month) or a nominal ($20/month) cash gift for the first several years of their children’s lives. Gifts were unconditional and mothers were informed they could use the money in any way they wished. The cash gifts increased income by an average of 20% in the large gift group. EEGs were recorded at 1 year in the infant’s home and results were adjusted for baseline characteristics.

The infants in the large gift group demonstrated increased power in the mid- to high-frequency bands (alpha, beta and gamma) compared with the nominal gift group, but there were no differences between groups in the low frequency bands.

*Comment*: Troller-Renfree et al. report 1-year results from the Baby’s First Years study. An unconditional cash gift of $333/month resulted in EEG changes that have been associated with better cognitive and behavioural outcomes. However, it should be noted that results did not survive correction for multiple comparisons. Nevertheless, this study is the first randomised controlled trial evidence of a causal relationship between SES and neurodevelopment. Furthermore, it suggests that fiscal interventions can improve cognitive and behavioural outcomes in infants of low-income families. The study also aims to report measured cognitive and behavioural outcomes at age four.

Troller-Renfree et al. (2022) Proc Natl Acad Sci USA 119(5):e2115649119.

